# Earlobe Blood Flow Responses to Different Intensities of Inspiratory Resistance Loading in Healthy Young Men

**DOI:** 10.7759/cureus.117904

**Published:** 2026-10-14

**Authors:** Yusuke Takahashi, Kazuki Okura, Daido Miyamoto, Kakeru Hasegawa, Ai Abe

**Affiliations:** 1 Department of Rehabilitation Medicine, Akita University Hospital, Akita, JPN

**Keywords:** circulation, healthy young man, inspiratory muscle training, respiratory muscle function, skin blood flow

## Abstract

Background and objective

Inspiratory resistance loading may alter peripheral circulation through changes in intrathoracic pressure and respiratory muscle pump activity. However, the relationship between inspiratory resistance intensity and peripheral blood flow remains unclear. This study aimed to investigate the response of earlobe blood flow (EBF) to varying intensities of inspiratory resistance loading in healthy young men.

Methods

Sixteen healthy young men performed multistep inspiratory resistance loading at 0%, 20%, 40%, 60%, and 80% of maximal inspiratory pressure (MIP) under incremental and decremental loading conditions on separate days. EBF was continuously measured, and the EBF ratio was calculated by dividing EBF at each loading intensity by resting EBF. The effects of loading intensity and loading condition were assessed using a linear mixed model. Changes in the EBF ratio across loading intervals were additionally evaluated using the slope (ΔEBF ratio/Δ% MIP).

Results

The EBF ratio increased significantly with increasing loading intensity (F(4,135) = 11.89). The EBF ratio was significantly higher at 40%, 60%, and 80% MIP than at 0% MIP, whereas no significant difference was observed between 0% and 20% MIP. Neither the loading condition nor its interaction with loading intensity was significant. The slope of the EBF ratio did not differ significantly among loading intervals (F(3,105) = 1.29).

Conclusion

Inspiratory resistance loading increased peripheral blood flow at low-to-moderate intensities, with the EBF response appearing to attenuate at approximately 40% MIP and above.

## Introduction

During exercise, an increase in cardiac output is required to maintain adequate blood flow to active muscles. The respiratory muscle pump is recognized as a circulatory mechanism that facilitates venous return through respiration-induced changes in intrathoracic pressure [1]. During inspiration, a reduction in intrathoracic pressure facilitates venous return from the peripheral circulation to the right heart, thereby contributing to circulatory support.

Inspiratory muscle training (IMT) improves inspiratory muscle strength and exercise capacity [2]. By enhancing the contractile strength of inspiratory muscles, including the diaphragm, IMT may reduce the work of breathing and improve hemodynamics through the respiratory pump effect [3]. Strengthening the respiratory muscle pump may have important physiological implications, potentially enhancing cardiac output by promoting venous return during exercise [1] and optimizing blood flow to peripheral tissues [4]. However, the effects of inspiratory resistance loading on peripheral circulation have not been sufficiently investigated, and the intensity of inspiratory resistance required to effectively elicit a circulatory assist effect remains unclear.

Earlobe blood flow (EBF) provides a noninvasive, continuous measure of peripheral blood flow and is stable to hemodynamic changes [5,6]. As a preliminary step toward investigating the potential circulatory effects of inspiratory resistance loading in patients with heart failure, it is important to first characterize the EBF response to varying inspiratory resistance levels in healthy individuals. Therefore, this study aimed to characterize the EBF response to varying intensities of inspiratory resistance loading in healthy young men using a multistep inspiratory resistance loading test. We hypothesized that the EBF response would increase with inspiratory resistance loading but that this increase would attenuate at loading intensities of 40% MIP or higher.

## Materials and methods

Participants

Healthy young men were recruited for this study. Men with a history of smoking, previous experience with IMT, apparent measurement errors, and inability to obtain clear visualization of the diaphragm by ultrasonography due to subcutaneous fat were excluded. The a priori sample size calculation was performed using G Power, assuming an effect size of 0.30, a significance level of α = 0.05, and a statistical power of 80% (β = 0.20). The effect size was selected based on conventional benchmarks. The calculation indicated that 15 participants were required. The target sample size was set at 16 to allow equal allocation of participants to the two loading sequences.

This study conformed to the Declaration of Helsinki (2024), the Ethical Guidelines for Medical and Biological Research Involving Human Subjects, and the Act on the Protection of Personal Information. Ethical approval was obtained from the Ethics Committee of the first author's affiliated institution (approval number: 3285). Written informed consent was obtained from all participants after the study procedures had been explained.

Procedure

This study was a prospective observational study. A multistep inspiratory resistance loading test based on maximal inspiratory pressure (MIP) was performed to assess the EBF response to inspiratory resistance loading. Inspiratory resistance was set at 0%, 20%, 40%, 60%, and 80% of MIP. The experimental setup is shown in Figure 1A, and the earlobe blood flow measurement site is shown in Figure 1B. Participants sat on a chair with a backrest, maintaining an upright and stable trunk without leaning against the backrest. The knees were slightly flexed, and the legs were kept uncrossed. Participants were instructed to maintain the same posture throughout the test. Representative raw data of earlobe blood flow is presented in Figure 1C. Inspiratory resistance loading consisted of incremental and decremental loading tests, during which the load pressure was progressively increased and decreased, respectively. The order of the two tests was balanced across participants to account for potential order effects, including carryover effects. Each test was performed on a separate day, and the order of the tests was randomized for each participant (Figure 2). A digital respiratory muscle trainer (POWERbreathe KH2; POWERbreathe International Ltd., Warwickshire, England) was used to apply the inspiratory resistance load. The device recognizes and records only inspirations that reach the preset inspiratory pressure as valid measurements. Participants were instructed to inhale as rapidly as possible to maximal inspiration and then exhale to maximal expiration. This instruction was used to standardize the respiratory maneuver and minimize variability in the degree of expiration before each inspiratory maneuver across participants and loading levels. Before the inspiratory resistance loading protocol, baseline measurements of body composition and diaphragmatic thickness were obtained.

**Figure 1:**
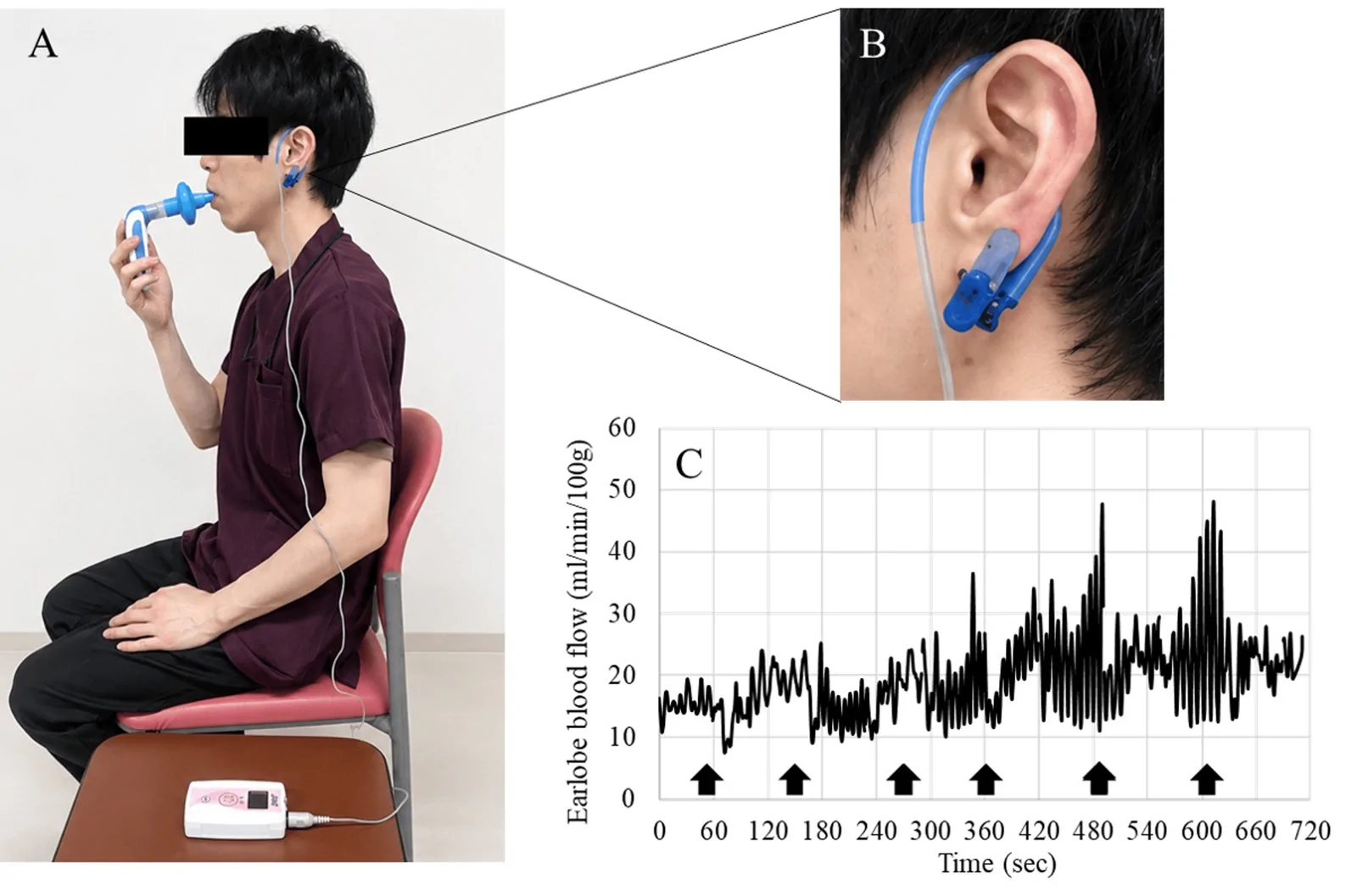
Measurement setting (A) Experimental setup during inspiratory resistive loading. Participants sat on a chair with a backrest, maintaining an upright and stable trunk without leaning against the backrest. The knees were slightly flexed, and the legs were kept uncrossed. Participants were instructed to maintain the same posture throughout the test. (B) Magnified view of the earlobe blood flow measurement site. (C) Representative continuous earlobe blood flow (EBF) recording from one participant during the incremental inspiratory resistance loading protocol. Inspiratory resistance was increased sequentially from 0% to 20%, 40%, 60%, and 80% of maximal inspiratory pressure (MIP). At each loading level, EBF was quantified as the mean value during the final 10 s of the corresponding stage (black arrows).

**Figure 2:**
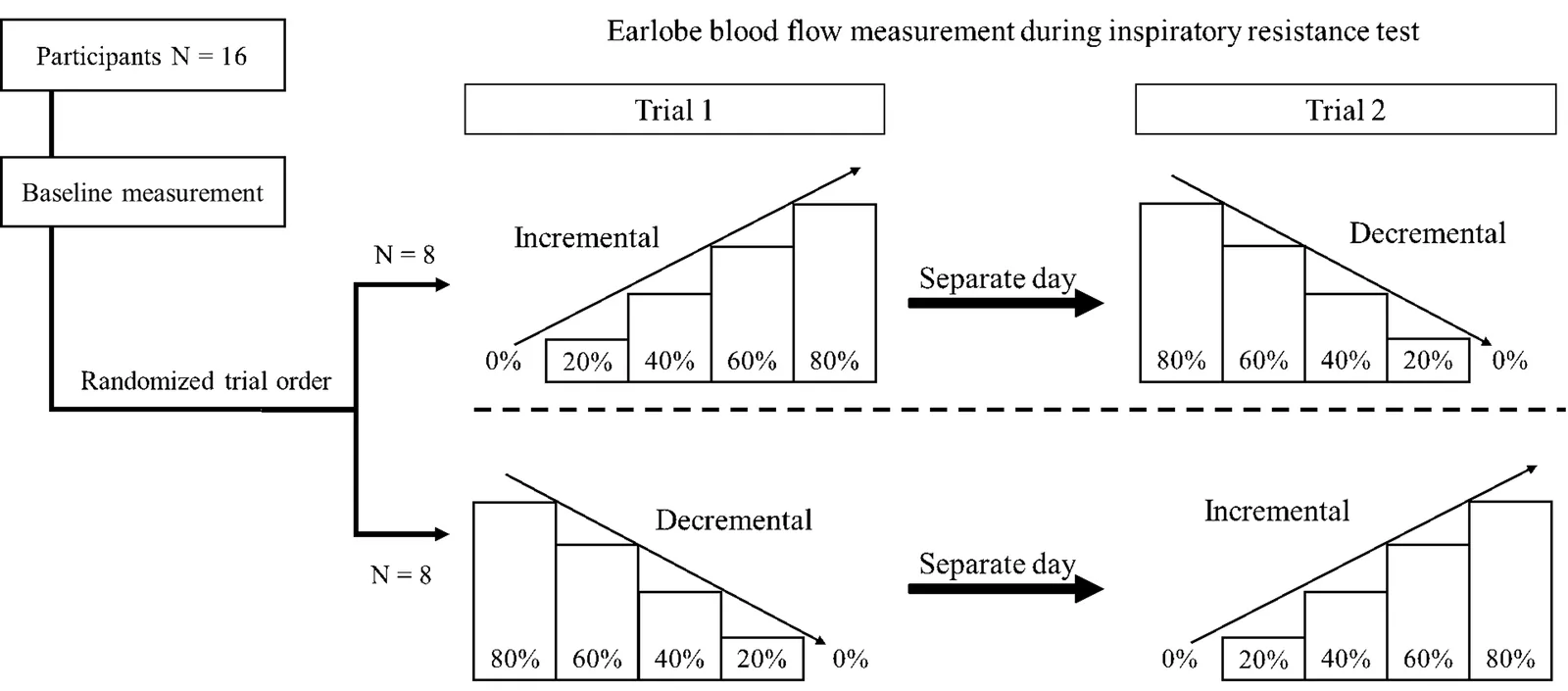
Procedure A multistep inspiration resistance loading test based on maximal inspiratory pressure (MIP) was performed to assess the EBF response to inspiratory resistance loading. Inspiratory resistance was set at 0%, 20%, 40%, 60%, and 80% of MIP. Inspiratory resistance loading consisted of incremental and decremental loading tests, during which the load pressure was progressively increased and decreased, respectively. Each test was performed on a separate day, and the order of the tests was randomized for each participant.

Maximal inspiratory pressure (MIP)

MIP was measured using the same digital respiratory muscle trainer. A nose clip was applied, and participants were instructed to perform a maximal inspiratory effort from maximal expiration while seated at rest. Three measurements were obtained, and the highest value was used for analysis.

Earlobe blood flow (EBF)

EBF was measured using a wireless laser Doppler blood flow meter (Pocket LDF; JMS, Tokyo, Japan). A clip-type sensor was attached to each participant's earlobe. The sampling frequency of the EBF waveform was set at 50 Hz. Measurement data were transmitted via Bluetooth and recorded on a personal computer equipped with custom-developed data acquisition software. Measurements were performed with participants in a seated position under a constant room temperature. Participants were instructed to refrain from caffeine intake and strenuous physical activity on the day of measurement. Before the inspiratory resistance loading protocol, participants rested for 5 min. Baseline EBF was defined as the mean EBF value recorded during the final 10 s of the resting period. Ten consecutive inspirations were performed at each inspiratory resistance load, and the mean EBF value during the final 10 s of each loading period was used as the representative value for that load.

Diaphragm thickness (Tdi)

Diaphragm thickness (Tdi) was measured using an ultrasound imaging device (SONIMAGE MX1, Konica Minolta, Inc., Tokyo, Japan) with participants in the resting supine position. Using B-mode ultrasonography, a probe coated with ultrasound gel was placed on the body surface at the eighth or ninth intercostal space between the right midaxillary and anterior axillary lines, corresponding to the zone of apposition, where the diaphragm was considered to be in contact with the rib cage. The probe position was finely adjusted to obtain clear visualization of the Tdi boundaries between the pleural and peritoneal layers, and ultrasound videos were recorded during resting expiration and maximal inspiration. In the recorded videos, frames showing the minimum Tdi were defined as the end of resting expiration, whereas frames showing the maximum Tdi were defined as maximal inspiration. Still images of these frames were extracted, and Tdi was measured to the nearest 0.1 mm using the built-in caliper function.

Tdi was measured three times, and the highest value was used for analysis. The Tdi change ratio (Tdi%) was calculated from the measured Tdi at resting expiration (Tdi min) and maximal inspiration (Tdi max) using the following equation:

$Tdi\% = \frac{Tdi_{max}-Tdi_{min}}{Tdi_{min}}\times100$

Body composition

Body composition was assessed using a multi-frequency bioelectrical impedance analysis (BIA) device (InBody S10, InBody Japan). Measurements were performed with participants standing barefoot and with all accessories and metal objects removed from the body surface. During measurement, the feet were placed on the designated electrodes, and the thumbs and fingers of both hands were placed in contact with the clip electrodes. Participants were instructed to keep their upper limbs slightly abducted from the trunk and maintain a position without contact between the trunk and upper limbs or between the body and other surfaces. Skeletal muscle mass, body fat mass, fat-free mass, and body fat percentage were determined.

Statistical analysis

The EBF ratio was calculated as EBF at each inspiratory resistance load divided by resting EBF and used for analysis to account for interindividual variability in resting blood flow.

To examine the relationship between inspiratory resistance loading intensity and EBF ratio, a linear mixed model (LMM) was used. The EBF ratio was specified as the dependent variable, with loading intensity (0%, 20%, 40%, 60%, and 80% MIP) and loading condition (incremental and decremental), and their interaction included as fixed effects. Participant ID was included as a random effect with a random intercept to account for interindividual variability. The model was estimated using restricted maximum likelihood, and the significance of the fixed effects was assessed using a type III analysis of variance with the Satterthwaite approximation. Because the interaction between loading intensity and loading condition was not statistically significant (p = 0.069), estimated marginal means (EMMs) were calculated for each loading intensity, averaged across the incremental and decremental conditions. Pairwise comparisons between loading intensities were then performed with Bonferroni adjustment.

To examine whether the rate of increase in EBF ratio differed across loading intervals, the slope of EBF ratio (ΔEBF ratio/Δ%MIP) was calculated for each participant for the 0-20%, 20-40%, 40-60%, and 60-80% MIP intervals. An LMM was then used with the calculated slope as the dependent variable to evaluate differences in slope among loading intervals.

All statistical analyses were performed using R version 4.5.2 (R Foundation for Statistical Computing, Vienna, Austria). The lme4 and lmerTest packages were used for LMM analyses, and the emmeans package was used to calculate the EMMs. Statistical significance was set at p < 0.05.

## Results

Sixteen healthy young men participated in the study. The baseline characteristics, Tdi, and MIP are presented in Table 1. All participants performed 10 inspiratory maneuvers at each loading intensity, with all 10 inspirations reaching the preset inspiratory pressure at 20%, 40%, and 60% MIP. At 80% MIP, all participants also performed 10 inspiratory maneuvers; however, all 10 inspirations reached the preset pressure in only five participants (31.3%) during the incremental loading test and six participants (37.5%) during the decremental loading test. Because EBF was measured continuously throughout each loading period, the EBF value at 80% MIP was calculated as the mean EBF during the final 10 s of the loading period, regardless of the number of inspirations that reached the preset pressure.

**Table 1:** Participant characteristics (n = 16) Data are presented as mean and standard deviation (SD). Tdi, diaphragm thickness; ΔTdi, difference between inspiratory and expiratory diaphragm thickness; MIP, maximal inspiratory pressure

Parameters	Unit	Mean	SD
Age	Years	21.0	1.4
Height	cm	173.5	4.4
Weight	kg	64.4	5.7
Skeletal muscle index	kg/m^2^	8.9	0.5
Phase angle	°	6.6	0.6
Tdi			
Inspiration	mm	4.1	1.3
Expiration	mm	1.8	0.6
ΔTdi	mm	2.3	0.8
MIP			
Incremental trial	cmH_2_O	83.9	16.8
Decremental trial	cmH_2_O	90.9	21.8

The EBF ratio changed with increasing inspiratory resistance intensity in both the incremental (Figure 3A) and decremental (Figure 3B) loading conditions. The results of the LMM analysis are presented in Table 2. The LMMs revealed a significant main effect of loading intensity (F(4, 135) = 11.89, p < 0.001), indicating that the EBF ratio differed across loading intensities. By contrast, the main effect of loading condition (incremental vs. decremental) was not significant (F(1, 135) = 0.873, p = 0.352). The interaction between loading intensity and loading condition was also not significant (F(4, 135) = 2.23, p = 0.069).

**Figure 3:**
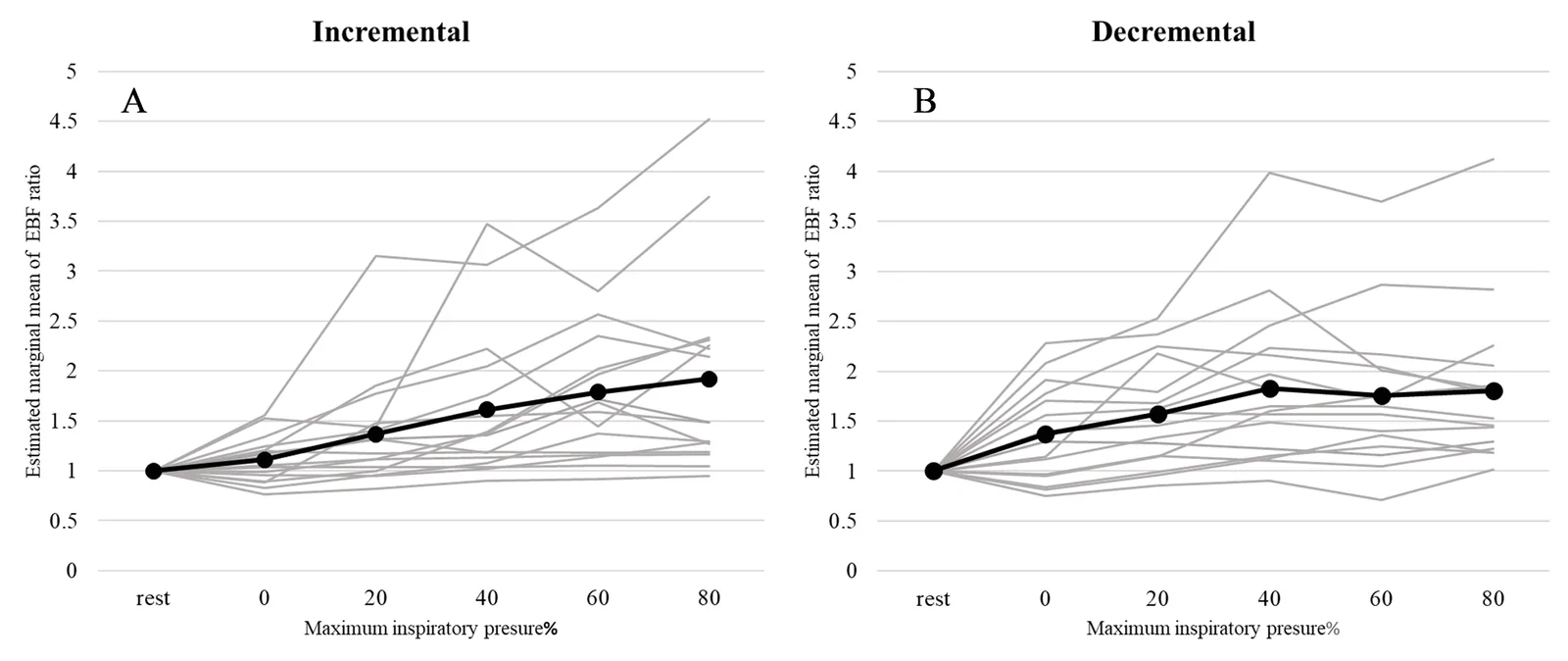
Changes in earlobe blood flow (EBF) ratio across inspiratory resistance loads Gray lines represent individual participants (n = 16), whereas the black line with circles represents the group mean. The left (A) and right (B) panels show the incremental and decremental loading conditions, respectively. The EBF ratio was calculated as EBF at each inspiratory resistance load divided by resting EBF.

**Table 2:** Effects of the inspiratory load and loading condition on the EBF ratio Results of a linear mixed model with loading condition and inspiratory load as fixed effects and participant as a random effect. Loading condition indicates incremental versus decremental loading, and load indicates inspiratory resistance intensity expressed as a percentage of maximal inspiratory pressure. NumDF, numerator degrees of freedom; DenDF, denominator degrees of freedom; EBF, earlobe blood flow

Effect	NumDF	DenDF	F	p
Condition	1	135	0.873	0.3518
Load (%)	4	135	11.888	<0.001
Condition × Load (%)	4	135	2.227	0.0694

Post-hoc pairwise comparisons of the EMMs showed that the EBF ratio was significantly higher at 40%, 60%, and 80% MIP than at 0% MIP (0% vs. 40%, p < 0.001; 0% vs. 60%, p < 0.001; 0% vs. 80%, p < 0.001). By contrast, the EBF ratio did not differ significantly between 0% and 20% MIP (p = 0.48). Among loading intensities of 40% MIP or higher, the EBF ratio did not differ significantly between 40% and 60% MIP (p = 1.00), 40% and 80% MIP (p = 0.352), or 60% and 80% MIP (p = 1.00). The EMMs of the EBF ratio are shown in Figure 4.

**Figure 4:**
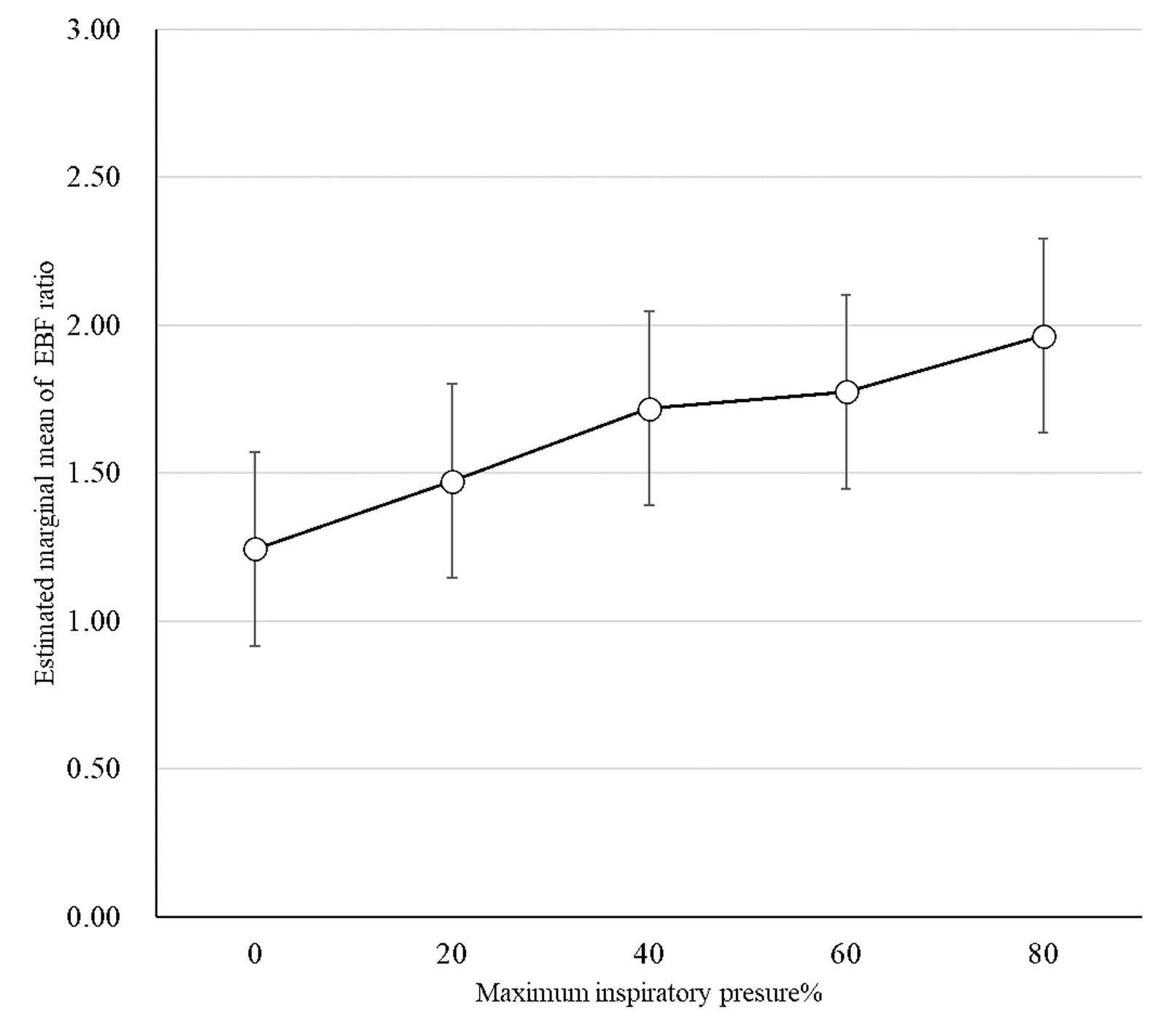
Estimated marginal means of the EBF ratio across the inspiratory loads Points indicate estimated marginal means derived from the linear mixed-effects model. The error bars represent 95% confidence intervals. The EBF ratio was significantly higher at 40%, 60%, and 80% MIP than at 0% MIP (all p < 0.01).

Furthermore, the LMM showed no significant difference in slope among the loading intervals (F(3, 105) = 1.29, p = 0.281). The interaction between loading interval and loading condition was not significant (F(3, 105) = 0.90, p = 0.445). Post-hoc pairwise comparisons revealed no significant differences among the loading intervals (adjusted p > 0.05).

## Discussion

This study investigated the changes in EBF in response to multistep respiratory resistance loading in healthy young men. The EBF ratio increased with respiratory resistance loading and was significantly higher at 40%, 60%, and 80% MIP than at 0% MIP. By contrast, no significant differences were noted between 40%, 60%, and 80% MIP. The EBF ratio did not exhibit any further increase above 40% MIP. The change in the EBF ratio across loading intensities did not differ significantly between the incremental and decremental loading conditions. These findings suggest that the EBF response to inspiratory resistance loading may increase at low-to-moderate loading intensities and then show a more gradual increase from approximately 40% MIP.

Possible explanations for the EBF response to inspiratory resistance loading

The increase in EBF observed during inspiratory resistance loading may be associated with changes in venous return related to the respiratory muscle pump and a potential increase in the tissue perfusion pressure gradient through the siphon effect. However, these mechanisms were not directly assessed in the present study and should therefore be interpreted as possible physiological explanations for the observed EBF response.

The respiratory muscle pump generates blood flow from the abdomen to the thoracic cavity through changes in intrathoracic pressure, thereby facilitating venous return and contributing substantially to the return of blood to the heart during upright activity [1]. In the present study, increased respiratory muscle pump activity associated with inspiratory resistance loading may have enhanced venous return, resulting in hemodynamic changes that contributed to the increase in EBF. In addition, decreased intrathoracic pressure during inspiration may affect perfusion of the head, including the earlobe, through the siphon effect, which facilitates blood movement toward the thoracic cavity. The siphon effect is a phenomenon in which negative intrathoracic pressure during inspiration promotes venous return, lowers intracranial pressure, and increases the tissue perfusion pressure gradient [3]. In particular, cerebral blood flow, which is less affected by abdominal pressure than blood flow in other regions [1], may be more susceptible to the siphon effect, as negative intrathoracic pressure facilitates blood flow through the capillary bed [3]. From 0% to 40% MIP, increasing inspiratory effort may have progressively increased negative intrathoracic pressure, thereby enhancing circulatory responses mediated by the respiratory muscle pump and siphon effect and contributing to the increase in EBF.

Attenuation of the EBF response above 40% MIP

By contrast, the EBF ratio did not increase significantly from 40% to 80% MIP. The attenuation of the increase in the EBF ratio above 40% MIP may be explained by limitations in diaphragmatic activity, as the diaphragm is the primary inspiratory muscle. Liu et al. reported that diaphragmatic activity increases proportionally with increasing inspiratory load but reaches a peak at approximately 50% MIP and subsequently decreases, indicating a nonlinear response [7]. Furthermore, at loading intensities above 50% MIP, the activity of accessory inspiratory muscles, such as the sternocleidomastoid muscle, becomes predominant over diaphragmatic activity. Therefore, the attenuation of the increase in the EBF ratio observed above 40% MIP may reflect a limited increase in respiratory muscle pump activity resulting from a plateau in diaphragmatic activity and a shift in the relative contribution of inspiratory muscles toward accessory muscles at higher loading intensities. However, because diaphragmatic activity was not directly assessed during inspiratory loading in the present study, these proposed mechanisms remain speculative.

By contrast, at 80% MIP, only five participants in the incremental loading test and eight participants during the decremental loading test achieved 10 valid inspirations that reached the preset inspiratory pressure. The respiratory muscle trainer used in this study was designed to open the valve only when the preset inspiratory pressure was reached. Therefore, inspiration that failed to reach the preset pressure may have involved strong inspiratory efforts against an occluded airway. This situation may resemble the Mueller maneuver, in which a strong inspiratory effort against an occluded airway generates markedly negative intrathoracic pressure. The Mueller maneuver is an experimental procedure primarily used to assess upper airway obstruction and sleep apnea and affects hemodynamics, including venous return, cardiac preload, and arterial pressure, through the generation of markedly negative intrathoracic pressure [8,9]. Therefore, at 80% MIP, changes in intrathoracic pressure associated with strong inspiratory efforts, in addition to the normal respiratory muscle pump effect, may have influenced the EBF response.

Clinical implication

Exercise therapy for patients with heart failure has traditionally focused primarily on improving peripheral skeletal muscle function to enhance exercise capacity. More recently, the effectiveness of exercise programs incorporating IMT has also been reported [2]. However, studies investigating the effects of IMT on hemodynamics, particularly its influence on the circulatory system through the respiratory pump mechanism, remain limited. IMT is also one of the few physical therapy interventions that can be implemented in patients with severe heart failure. The relationship between inspiratory loading intensity and EBF response demonstrated in the present study provides foundational evidence for re-examining the physiological effects of IMT from a circulatory perspective and may help inform the development of novel exercise therapy strategies for patients with heart failure.

Limitations

Several limitations of this study should be acknowledged. First, the ability to achieve the preset inspiratory pressure decreased at higher loading intensities. At 80% MIP, only a limited number of participants achieved the preset pressure in all 10 inspirations, limiting the number of valid inspirations. Therefore, the EBF responses at higher loading intensities, particularly at 80% MIP, should be interpreted with caution. Second, the sample size was relatively small, and the study included only 16 healthy young men. This restricted study population limits the generalizability of the findings to women, older adults, and patients with cardiovascular or respiratory diseases. Further studies involving larger and more diverse populations, including relevant patient populations, are needed to determine whether similar EBF responses occur under clinical conditions. Third, hemodynamic variables, including intrathoracic pressure, venous return, and cardiac output, were not directly measured. Therefore, the physiological mechanisms underlying the EBF response to inspiratory resistance loading could not be directly verified. Fourth, diaphragmatic activity and the occurrence of a Mueller maneuver-like condition during inspiratory loading were not directly assessed. Consequently, the contributions of changes in diaphragmatic activity and intrathoracic pressure associated with strong inspiratory efforts to the EBF response at higher loading intensities remain uncertain. Finally, the physiological interpretation of EBF requires clarification. Laser Doppler flowmetry primarily reflects local microvascular perfusion, as determined by the concentration and velocity of moving red blood cells, rather than directly measuring volumetric blood flow. Therefore, although the device reports EBF in mL/min/100 g and the EBF ratio was used to account for interindividual differences in resting values, the measured signal should not necessarily be interpreted as a direct measure of systemic peripheral blood flow.

## Conclusions

In healthy young men, the EBF ratio increased with increasing inspiratory resistance loading and was significantly higher at ≥40% MIP than at 0% MIP. By contrast, the EBF ratio did not increase significantly from 40% to 80% MIP, and the rate of increase did not differ significantly among loading intervals. These findings suggest that inspiratory resistance loading may increase earlobe microvascular perfusion at low-to-moderate loading intensities, with the response attenuation of the increase at higher intensities. The respiratory muscle pump and changes in intrathoracic pressure associated with inspiratory resistance may contribute to this response; however, further studies are needed to clarify the underlying mechanisms.
